# Efficacy and safety evaluation of rivaroxaban vs. warfarin among non-valvular atrial fibrillation patients undergoing lower extremity revascularization

**DOI:** 10.3389/fcvm.2022.978639

**Published:** 2022-09-07

**Authors:** Qingyuan Yu, Cheng Chen, Jinyan Xu, Yu Xiao, Junmin Bao, Liangxi Yuan

**Affiliations:** ^1^Department of Vascular Surgery, Changhai Hospital, Navy Military Medical University, Shanghai, China; ^2^ChangZheng Hospital, Navy Military Medical University, Shanghai, China

**Keywords:** oral anticoagulants, nonvalvular atrial fibrillation, peripheral arterial disease, lower extremity revascularization, revascularization

## Abstract

**Introduction:**

The efficacy and safety of antithrombotic strategies remain uncertain in patients with atrial fibrillation undergoing lower-extremity revascularisation.

**Materials and methods:**

Between January 2011 and November 2021, 319 patients with atrial fibrillation after lower-extremity revascularisation received rivaroxaban or warfarin treatment as anticoagulation regimens with different antiplatelet therapy strategies. The primary efficacy outcome was the composite of acute limb ischaemia, major amputation for vascular causes, myocardial infarction, ischaemic stroke, clinically driven target lesion revascularisation, and death from vascular causes. The safety outcomes were major bleeding events according to the International Society on Thrombosis and Haemostasis classification criteria.

**Results:**

A total of 178 and 141 patients received rivaroxaban and warfarin treatments, respectively, after revascularisation with or without antiplatelet regimens. The incidence of the primary efficacy outcome at 36 months in the rivaroxaban group (44 patients, 24.7%) tended to be lower than that in the warfarin group (43 patients, 30.5%) (hazard ratio, 0.870; 95% confidence interval, 0.565–1.339; *P* = 0.527). The incidence of the secondary efficacy outcomes decreased in the rivaroxaban group (56 patients, 31.6%) compared with that in the warfarin group (61 patients, 43.2%). Major bleeding events occurred in three patients (1.7%) in the rivaroxaban group and five patients (3.5%) in the warfarin group; no significant difference in fatal or intracranial bleeding was observed between the groups.

**Conclusion:**

This study describes practical experience regarding the use of rivaroxaban and warfarin in patients with peripheral arterial disease complicated by non-valvular atrial fibrillation following endovascular intervention. The efficacy and safety outcomes do not differ significantly between rivaroxaban and warfarin.

## Introduction

The prevalence of atrial fibrillation (AF) in patients with peripheral arterial disease (PAD) is 10–13% (1, 2). AF and PAD have similar epidemiological patterns and risk factors that are associated with increased cardiovascular (CV) events and mortality. It has been reported that patients with AF and PAD have a higher incidence of adverse events; among them, the presence of PAD is significantly associated with a 1.3–2.5-fold increased risk of stroke, and the risk of thrombotic events, including ischaemic stroke, is increased up to 2-fold (3, 4).

The current guidelines recommend oral anticoagulant (OAC) therapy instead of antiplatelet therapy (APT) for patients with AF and PAD; meanwhile, the combination of OAC therapy and APT can be considered for patients with AF and PAD undergoing intravascular revascularisation (3, 5). However, OAC therapy combined with APT may increase severe bleeding, including intracranial bleeding (4, 6). To guide in the selection of OAC therapy, few studies have investigated the outcome of adverse limb events in patients with AF and concomitant PAD post-procedure receiving OAC or APT regimens; further, it is uncertain whether new oral anticoagulants (NOACs) or warfarin is more effective (7, 8).

Therefore, the aim of this study was to investigate the efficacy and safety outcomes of NOACs compared with those of warfarin in patients with AF and concomitant PAD following endovascular intervention.

## Materials and methods

This single-centre retrospective study included all sequential patients who were prescribed rivaroxaban or warfarin after endovascular intervention for chronic lower-extremity arterial occlusive disease or acute embolic thrombus occlusion with concomitant non-valvular atrial fibrillation (NVAF) between January 2011 and November 2021. Patient demographics, comorbidities, lesion characteristics, pre-procedural medications, CHA_2_DS_2_-VASc score, HAS-BLED score, and procedural details were recorded. The comorbidities included hypertension, diabetes, smoking-related conditions, coronary artery disease, ischaemic stroke, and chronic renal failure.

Patients were excluded when they had significant haemorrhagic transformation, mechanical/prosthetic heart valves, haemodynamically significant mitral stenosis, end-stage renal disease, or a recent stroke or systemic embolic event or were at risk of bleeding or switching between two anticoagulants postoperatively.

### Definitions

The CHA_2_DS_2_-VASc score (age of 75 years or above, 2 points; previous stroke or transient ischaemic attack, 2 points; congestive heart failure, hypertension, diabetes, vascular disease, age of 65–74 years, and female sex, 1 point) was calculated to quantify the risk of thromboembolic events in the patients with AF. The HAS-BLED score [hypertension, renal or liver dysfunction, stroke, history of bleeding, unstable international normalised ratio (INR), age of 65 years or older, antiplatelet drug use, or alcohol use] was calculated to assess the bleeding risk in the patients with AF treated with OACs (9, 10).

#### Periprocedural anticoagulation regimen

Rivaroxaban was prescribed at a dose of 10 mg, once daily. All patients in the warfarin group were bridged with unfractionated or low-molecular-weight heparin periprocedurally, and their INR was maintained between 2 and 3. The patients were required to take the planned rivaroxaban/warfarin dose and continue antiplatelet drug use after surgery.

### Outcomes

#### Efficacy outcomes

The primary efficacy outcome was the composite of acute limb ischaemia, major amputation for vascular causes, myocardial infarction, ischaemic stroke, clinically driven target lesion revascularisation (CD-TLR), or death from vascular causes. The secondary efficacy outcomes included the composite of acute limb ischaemia, major amputation for vascular causes, myocardial infarction, ischaemic stroke, CD-TLR, or death from any cause. The composite of major adverse limb events included acute limb ischaemia, major amputation for vascular causes, and CD-TLR.

#### Safety outcomes

The primary safety outcome was major bleeding defined by the International Society on Thrombosis and Haemostasis as intracranial or severe bleeding that was sufficient to result in death, surgery, cessation of therapy, dropping of the haemoglobin level to 2.0 g/dL, or transfusion of 2 units of blood (11, 12). Gastrointestinal bleeding was assessed as the secondary safety outcome.

#### Data collection

Outpatient monitoring of INR was conducted once every week of discharge and the dose of warfarin was adjusted until the value achieve and maintain the therapeutic INR between 2 and 3. All patients were followed up within 30 days after surgery and planned to return every 3 months in the first year after treatment and every 6 months thereafter. Preoperative and postoperative evaluation data included clinical manifestations, symptoms, complications, anticoagulant use, and ultrasound findings. Follow-up imaging was mainly performed using dual-function ultrasound scanning. Computed tomography angiography was performed when symptoms recurred, or more than 50% restenosis was detected on Doppler ultrasound. The clinical secretary conducted a monthly telephone follow-up to assess the incidence of bleeding. The follow-up period was defined as 3 years of discharge or the end date of the study period (31*^st^* May 2022), whichever occurred first.

### Statistical analyses

All analyses were performed using the SPSS software (version 26.0, Chicago, IL, United States). Continuous data were expressed as means ± standard deviations, categorical data as numbers and percentages, and non-normally distributed data as medians and interquartile ranges. Differences between the two cohorts were compared using the chi-square test and Fisher’s exact test for categorical variables and the *t*-test for continuous variables. Statistical significance was set at *P* < 0.05. The event probability was expressed as a Kaplan–Meier estimate of the 3-year cumulative incidence. Factors identified in the univariate analysis (*P* < 0.3) and other variables considered likely to have important prognostic values were tested in the multivariate Cox proportional hazard model, which was used to generate hazard ratios (HRs) and 95% confidence intervals (CIs) (Supplementary Tables 1, 2).

## Results

### Baseline characteristics

The baseline characteristics were well balanced between the groups (Table 1). The median patient age was 80 years, and 47% of the patients were women. Approximately one-third of the patients had atherosclerosis obliterans (39.8%), and two-thirds underwent thrombus embolisation (60.2%). The risk factors were common: 26.3% of the patients had diabetes mellitus; 26.3% had chronic renal failure; and 19.4% were smokers. Approximately 31% of the patients had a previous ischaemic stroke; 30.1% had a previous coronary artery disease; and 69.6% had hypertension. The CHA_2_DS_2_-VASc and HAS-BLED scores were higher in the rivaroxaban group than in the warfarin group. Approximately 87.8% of the patients had *de novo* lesions, and 62.7% had lesions > 10 cm in length. A total of 55 patients (17.2%) underwent index revascularisation for critical limb ischaemia, and 67.4% underwent thrombus debulking. In terms of pre-procedural medications, 26% received single APT, while 3.8% received dual APT. Post-procedurally, without accounting for anticoagulant treatments, 82.4% received single APT, while 17.6% received dual APT. The median clinical follow-up period was 36 months (interquartile range, 17.5–36 months).

**TABLE 1:** Baseline clinical characteristics of the patients.

Baseline characteristic	Total (*N* = 319)	Rivaroxaban (*N* = 178)	Warfarin (*N* = 141)	*P* Value
Median age, years	80.0 (71.0–84.0)	80.0 (66.0–94.0)	81.0 (69.0–93.0)	0.688
**Sex**				0.198
Male	169 (53.0%)	100 (56.2%)	69 (48.9%)	
Female	150 (47.0%)	78 (43.8%)	72 (51.1%)	
CHA2DS2-VASc score	5.07 ± 1.455	5.09 ± 1.478	5.04 ± 1.431	0.970
HAS-BLED score	2.28 ± 0.968	2.30 ± 0.984	2.25 ± 0.950	0.529
**Duration**				0.334
Acute	185 (58.0%)	99 (55.6%)	86 (61.0%)	
Chronic	143 (42.0%)	79 (44.4%)	55 (39.0%)	
**Diagnosis**				0.509
ASO	127 (39.8%)	68 (38.2%)	59 (41.8%)	
Thrombus Embolization	192 (60.2%)	110 (61.8%)	82 (58.2%)	
**Lesion characteristics**				0.935
*De Novo*	280 (87.8%)	156 (87.6%)	124 (87.9%)	
Restenosis	39 (12.2%)	22 (12.4%)	17 (12.1%)	
Lesion length				0.559
> 10cm	198 (62.1%)	113 (63.5%)	85 (60.3%)	
< 10cm	121 (37.9%)	65 (36.5%)	56 (39.7%)	
Thrombus Debulking	215 (67.4%)	121 (68.0%)	94 (66.7%)	0.804
Critical limb ischemia	248 (77.7%)	141 (79.2%)	107 (75.9%)	0.478
History of index-limb revascularization	55 (17.2%)	36 (20.2%)	19 (13.5%)	0.113
**Risk factors and coexisting conditions**				
Hypertension	222 (69.6%)	129 (72.5%)	93 (66.0%)	0.209
Diabetes mellitus	84 (26.3%)	42 (23.6%)	42 (29.8%)	0.212
Smoking status	62 (19.4%)	40 (22.5%)	22 (15.6%)	0.124
Coronary artery disease	99 (31.0%)	52 (33.3%)	47 (29.2%)	0.430
Ischemic stroke	96 (30.1%)	52 (29.2%)	44 (31.2%)	0.700
Chronic Renal failure	20 (6.3%)	11 (6.2%)	9 (6.4%)	0.941
**Pre-procedural medication**				0.467
No	224 (70.2%)	121 (68.0%)	103 (73.0%)	
Single antiplatelet	83 (26.0%)	51 (28.7%)	32 (22.7%)	
Dual antiplatelet	12 (3.8%)	6 (3.4%)	6 (4.3%)	
**Post-procedural medication**				0.266
Single antiplatelet	263 (82.4%)	143 (80.3%)	120 (85.1%)	
Dual antiplatelet	56 (17.6%)	35 (19.7%)	21 (14.9%)	

Data are shown as number (percentage), median (interquartile range) or mean ± SD.

### Efficacy outcomes

**FIGURE 1:**
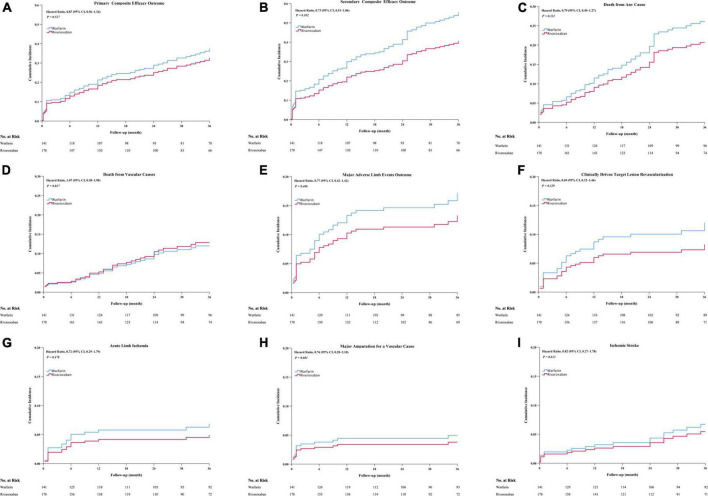
The cumulative incidence outcomes occurred between the rivaroxaban group and the warfarin group, including cumulative incidence of primary composite efficacy outcome **(A)**, secondary composite efficacy outcome **(B)**, death from all cause **(C)**, death from vascular cause **(D)**, major adverse limb events outcome **(E)**, CD-TLR **(F)**, acute limb ischemia outcome **(G)**, major amputation for a vascular cause **(H)**, Ischemia stroke **(I)**. CI, confidence intervals. CD-TLR, clinically driven target lesion revascularization. Major adverse limb events: Acute limb ischemia, major amputation for a vascular cause, or CD- TLR.

The rivaroxaban and warfarin groups did not differ significantly regarding the efficacy outcomes (Figure 1 and Table 2). The primary composite outcome occurred in 44 patients in the rivaroxaban group and 43 patients in the warfarin group, and the Kaplan–Meier estimates of the incidence at 3 years were 29.6% and 31.4%, respectively (HR, 0.87; 95% CI, 0.57–1.34; *P* = 0.527) (Figure 1A and Table 2). The incidence of the first secondary outcome was lower in the rivaroxaban group than in the warfarin group (HR, 0.73; 95% CI, 0.51–1.07; *P* = 0.102) (Figure 1B and Table 2); the Kaplan–Meier estimates of the incidence at 3 years were 35.5% and 43.6%, respectively. The all-cause mortality was lower in the rivaroxaban group than in the warfarin group (HR, 0.79; 95% CI, 0.49–1.27; *P* = 0.331); the Kaplan–Meier estimates of the incidence at 3 years were 22.3 and 27.9%, respectively (Figure 1C and Table 2). The incidence of vascular death was higher in the rivaroxaban group than in the warfarin group (HR, 1.07; 95% CI, 0.58–1.98; *P* = 0.817); the Kaplan–Meier estimates of the incidence at 3 years were 14.6% and 13.5%, respectively (Figure 1D and Table 2). The incidence of major adverse limb events was not lower in the rivaroxaban group than in the warfarin group (HR, 0.72; 95% CI, 0.42–1.42; *P* = 0.406); the Kaplan–Meier estimates of the incidence at 3 years were 15.3% and 17.1%, respectively (Figure 1E and Table 2). The rivaroxaban and warfarin groups did not differ significantly regarding the efficacy outcomes of CD-TLR, acute limb ischaemia, major amputation for vascular causes, or ischaemic stroke (Figures 1F–I and Table 2).

**TABLE 2:** Primary and secondary efficacy outcomes.

Outcome	Total (*N* = 319)	Rivaroxaban (*N* = 178)		Warfarin (*N* = 141)		Hazard Ratio (95% CI)	*P* Value
			
	Patients with event	Patients with event	K–M Estimate at 3 Yr	Patients with event	K–M Estimate at 3 Yr		
	No. (%)	No. (%)	%	No. (%)	%		
Primary efficacy outcome:acute limb ischemia, major amputation for vascular causes, myocardial infarction, ischemic stroke, CD- TLR or vascular death.	87 (27.3)	44 (24.7)	29.6	43 (30.5)	31.4	0.870 (0.565–1.339)	0.527
Acute limb ischemia	20 (6.3)	10 (5.6)	6.8	10 (7.1)	7.8	0.718 (0.287–1.794)	0.478
Major amputation for vascular causes	16 (5.0)	8 (4.5)	5.3	8 (5.7)	6.0	0.764 (0.279–2.096)	0.601
Myocardial infarction	8 (2.5)	5 (2.8)	3.6	3 (2.1)	2.3	1.848 (0.442–7.718)	0.400
Ischemic stroke	19 (6.0)	8 (4.5)	5.9	11 (7.8)	9.1	0.815 (0.370–1.797)	0.613
CD- TLR	30 (9.4)	15 (8.4)	10.1	15 (10.6)	11.9	0.688 (0.324–1.459)	0.329
Vascular death	38 (11.9)	20 (11.2)	14.6	18 (12.8)	13.5	1.074 (0.584–1.975)	0.817
**Secondary efficacy outcomes**							
Acute limb ischemia, major amputation for a vascular cause, myocardial infarction, ischemic stroke, CD- TLR or death from any cause	117 (36.7)	56 (31.6)	35.6	61 (43.2)	43.6	0.733 (0.506–1.064)	0.102
Major adverse limb events	45 (14.1)	23 (12.9)	15.3	22 (15.6)	17.1	0.772 (0.419–1.421)	0.406
Death from any cause	73 (22.9)	34 (19.1)	22.3	39 (27.7)	27.9	0.791 (0.494–1.268)	0.331

Data are shown as number (percentage). K–M denotes Kaplan–Meier. CI, confidence intervals. CD-TLR, clinically driven target lesion revascularization. Major adverse limb events: Acute limb ischemia, major amputation for a vascular cause, or CD- TLR.

**FIGURE 2:**
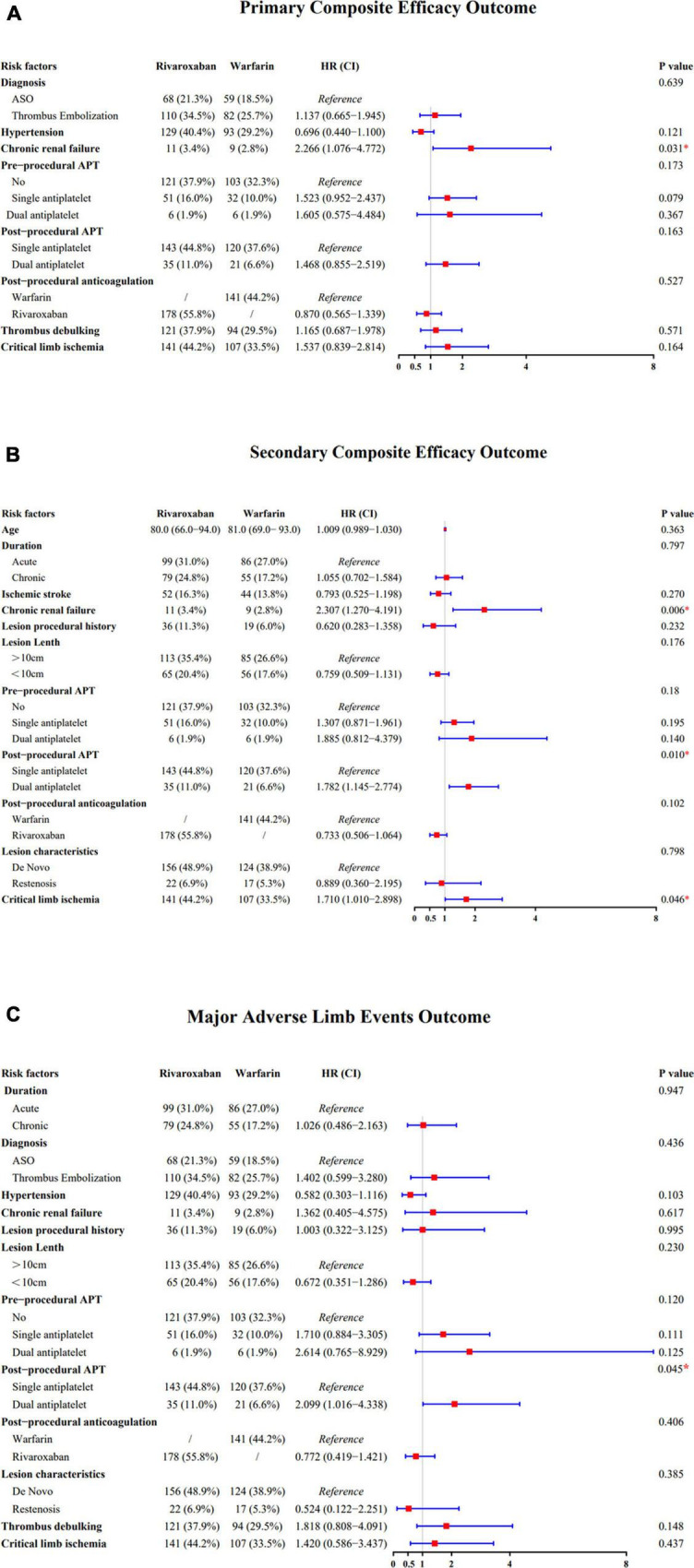
**(A)** Forest plot comparing the efficacy of the primary composite outcome across the risk factors. **(B)** Forest plot comparing the efficacy of the secondary composite outcome across the risk factors. **(C)** Forest plot comparing the efficacy of the major adverse limb events outcome across the risk factors.

Chronic renal failure increased the risk of the primary efficacy outcomes. There was no risk increase in the efficacy of the primary outcome across the other major risk factors, including those based on the diagnosis, critical limb ischemia, and hypertension risk factors (Figure 2A). Similarly, there was no risk increase in terms of pre-procedural APT, post-procedural APT, post-procedural anticoagulation, or thrombus debulking; meanwhile, there was significant risk increase in terms of chronic renal failure. There was also significant risk increase in the efficacy of the secondary outcomes of chronic renal failure, post-procedural APT, and critical limb ischemia (Figure 2B). Conversely, there was significant risk increase in terms of post-procedural APT (Figure 2C).

### Safety outcomes

The rivaroxaban and warfarin groups did not differ significantly regarding the safety outcomes. The primary safety outcome of major bleeding during follow-up occurred in three patients in the rivaroxaban group and five patients in the warfarin group, with Kaplan–Meier estimates of the incidence at 3 years of 2.6 and 3.7%, respectively (HR, 0.51; 95% CI, 0.12–2.07; *P* = 0.343) (Table 3). The composite outcome of intracranial or fatal bleeding occurred in two patients in each group (HR, 0.93; 95% CI, 0.121–7.13; *P* = 0.943). The secondary safety outcome of gastrointestinal bleeding occurred in four patients in the rivaroxaban group and two patients in the warfarin group; the Kaplan–Meier estimates of the incidence at 3 years were 2.6% and 1.7%, respectively (HR, 2.33; 95% CI, 0.42–12.82; *P* = 0.33) (Table 3).

**TABLE 3:** Safety outcomes.

Outcome	Total (*N* = 319)	Rivaroxaban (*N* = 178)		Warfarin (*N* = 141)		Hazard Ratio (95% CI)	*P* Value
			
	Patients with Event	Patients with Event	K–M Estimate at 3 Yr	Patients with Event	K–M Estimate at 3 Yr		
	No. (%)	No. (%)	%	No. (%)	%		
Principal safety outcome: ISTH major bleeding	8 (2.5)	3 (1.7)	2.6	5 (3.5)	3.7	0.506 (0.124–2.068)	0.343
Intracranial or fatal bleeding	4 (1.3)	2 (1.2)	1.7	2 (1.4)	1.4	0.929 (0.121–7.132)	0.943
**Secondary safety outcome**							
Gastrointestinal bleeding	6 (3.1)	4 (2.2)	2.6	2 (1.4)	1.7	2.325 (0.422–12.820)	0.333

Data are shown as number (percentage). K–M denotes Kaplan–Meier. CI, confidence intervals.

## Discussion

Among patients with PAD, those with AF are usually older than those with sinus rhythm, and most of them are complicated with diseases, such as hypertension, diabetes, chronic renal disease, coronary artery disease, and/or heart failure (13). According to the Rutherford classification, patients with AF have more severe PAD symptoms and a higher incidence of in-hospital complications, and PAD-related AF is an independent predictor of stroke, amputation, and death (2, 14).

However, in patients with AF undergoing lower-extremity revascularisation, antithrombotic strategies remain a challenge in clinical practice. The risk of ischaemic and haemorrhagic events must be carefully balanced (15). In patients with AF and PAD, there is a significant 56% reduction in the incidence of acute limb events when receiving rivaroxaban compared with that when receiving warfarin (1), and current clinical practice is more inclined to the use of NOACs, such as rivaroxaban. The following advantages of rivaroxaban should be noted: no temporary hypercoagulable state, stable anticoagulation effect, fewer drug–food or drug–drug interactions, and less unnecessary INR monitoring to adjust the dose (8, 16, 17). Previous studies have indicated that rivaroxaban affects protease-activated receptors to inhibit cell signalling in atrial myocytes or endothelial cells, thus playing an important role in the pro-inflammatory response to prevent related adverse events (8, 18).

The current guidelines for the optimal dose of rivaroxaban when considering efficacy and safety are based on global trial results (19). Most NOAC trials included a low proportion of Asian participants, such as 6.5% in the ROCKET-AF trial (19). To date, several studies have focused on the issue of reduced rivaroxaban doses in Asian populations. Asians are more prone to anticoagulant-related and intracranial bleeding than Caucasians owing to differences in race and lifestyle (15). Another study showed that in healthy Chinese individuals, 10 mg rivaroxaban may be sufficient to reach 83% of inhibition of factor Xa activity caused by a 20-mg dose (17). Furthermore, the Korean Heart Rhythm Society set 75–80 years of age as the standard age for rivaroxaban dose reduction (19). Thus, off-label rivaroxaban dose reduction is a common clinical practice in Asia.

In our research, the dose of rivaroxaban administered to the patients was 10 mg per day, and the dose reduction was mainly attributed to the following: (i) The median age of the patients in our cohort was 80.0 years (range, 71.0–84.0 years), which is higher than those in AF registry trials (e.g., 73 years in the ROCKET-AF trial; 71.5 years in the XANTUS trial). (ii) For these patients, renal creatinine clearance probably declines, and the time for rivaroxaban to be metabolised in the body will be prolonged (18, 20, 21). (iii) More importantly, a considerable number of patients in our cohort required single -antiplatelet or dual APT regimens postoperatively. Considering that standard doses may increase the risk of bleeding in patients, a reduced dose of 10 mg rivaroxaban per day for patients with AF who have undergone lower-extremity revascularisation is appropriate in clinical practice.

Herein, we also compared the efficacy and safety of rivaroxaban with those of warfarin in the patients with AF who underwent lower-extremity revascularisation using the Cox proportional hazard model. Similar to other studies, our study revealed a non-significant trend toward an overall lower incidence of the primary composite efficacy outcome in the rivaroxaban group than in the warfarin group. Although there was no significant difference between the two groups, rivaroxaban was associated with a reduced risk of adverse limb events. In terms of the secondary efficacy outcomes, our study also demonstrated a similar result for rivaroxaban. In the ROCKET-AF trial, rivaroxaban has not been reported to be related to a significantly higher risk of stroke or systemic embolism than warfarin (22). Lee et al. noted that NOACs were associated with a similar risk of ischaemic stroke and a reduced risk of acute myocardial infarction, major adverse limb events, and major bleeding events (3). Compared with the incidence in these previous studies, the high incidence of systemic embolism or vascular death in our study is probably attributed to the following: an older age (median age: 80.0 years in our study vs 73 years in the ROCKET-AF trial); a higher incidence of concomitant coronary or cerebral artery diseases; a higher CHA_2_DS_2_-VASc score (warfarin group: 5.04 ± 1.43 in our study vs 4.43 ± 1.65 in the study by Lee et al.; rivaroxaban group: 5.09 ± 1.48 in our study vs 4.41 ± 1.67 in the study by Lee et al.). Moreover, our research focused on patients with PAD requiring endovascular procedures rather than a broad population of patients with PAD. Taken together, rivaroxaban has the advantage of reducing the risk of composite efficacy outcomes. In our subgroup analysis, although not significant, the advantage of rivaroxaban over warfarin persisted in reducing the risks of acute limb ischaemia, major amputation for vascular causes, revascularisation for recurrent limb ischaemia, and all-cause death.

Another major finding was that low-dose rivaroxaban was non-inferior to warfarin in terms of the primary safety outcomes, including major and intracranial or fatal bleeding. The incidence of gastrointestinal bleeding was slightly higher in the rivaroxaban group than in the warfarin group. No difference in the overall bleeding events was observed between the rivaroxaban and warfarin groups during the follow-up period, with only 1.2% of the patients experiencing fatal or intracranial bleeding and 2.2% experiencing gastrointestinal bleeding in the rivaroxaban group. Contrary to our study, the ROCKET-AF trial demonstrated that rivaroxaban yielded a higher bleeding risk than did warfarin but also reported that the excessive bleeding events with rivaroxaban were the result of non-fatal mucosal bleeding. We hypothesised that the different opinions regarding haemorrhagic safety events in current studies may be related to the different bleeding definitions used by investigators. The incidence of fatal and intracranial bleeding, which required a specific focus, is similar in each study. Available evidence suggests that peri-procedural measures of anticoagulation or antiplatelet regimens and use of PPI, glycoprotein IIb/IIIa inhibitors, and other factors may be considered to further prevent bleeding (23).

In our study, 82.4% of all patients received single APT, while 17.6% received dual APT. Triple therapy has been widely demonstrated to cause an increase in the incidence of bleeding events, with no apparent benefit in the prevention of postoperative restenosis and systemic thrombosis. In the VOYAGER PAD study, the efficacy and safety of dual-pathway inhibition regimens were consistent with those of aspirin. The addition of clopidogrel did not further reduce the risk of limb and CV events, whereas its combination increased the risk of bleeding. This also provides support for postoperative drug use in patients with AF who have undergone lower-extremity revascularisation.

### Limitations

This study was a retrospective analysis with a relatively small sample size. Further randomised and prospective studies are necessary to evaluate limb prognosis in patients with AF and concomitant PAD treated with NOACs and warfarin. Additionally, no further subgroup analysis was conducted, and the heterogeneity results of the main subgroups need to be further verified.

## Conclusion

This study describes practical experience regarding the use of rivaroxaban and warfarin in patients with PAD complicated by NVAF following endovascular intervention. The efficacy and safety outcomes do not differ significantly between rivaroxaban and warfarin.

## Data availability statement

The original contributions presented in this study are included in the article/Supplementary material, further inquiries can be directed to the corresponding author.

## Author contributions

LY: conception and design, analysis and interpretation, critical revision, approval of the manuscript, agreement to be accountable, statistical analysis, and obtaining funding. QY: data collection, analysis and interpretation, writing the manuscript, critical revision, approval of the manuscript, agreement to be accountable, and statistical analysis. CC: conception and design, writing the manuscript, critical revision, approval of the manuscript, agreement to be accountable, and statistical analysis. JX: analysis and interpretation, writing the manuscript, approval of the manuscript, and agreement to be accountable. YX: analysis and interpretation, critical revision, approval of the manuscript, and agreement to be accountable. JB: conception and design, critical revision, approval of the manuscript, and agreement to be accountable. All authors contributed to the article and approved the submitted version.
