# Supplementary material for: Efficacy and safety evaluation of rivaroxaban vs. warfarin among non-valvular atrial fibrillation patients undergoing lower extremity revascularization
Source: Front Cardiovasc Med. 2022 Sep 7;9:978639. doi: 10.3389/fcvm.2022.978639 (PMC9489830; doi:10.3389/fcvm.2022.978639)
Supplement: Supplementary file 1 [file Table_1.docx]

**SUPPLEMENTARY MATERIAL**

**Results**

Factors identified in the univariate analysis (P < 0.3) and other variables considered likely to have important prognostic values were tested in the multivariate Cox proportional hazard model.

Diagnosis, thrombus debulking, critical limb ischemia,hypertension, chornic renal failure and post-procedural anticoagulant were entered into the multivariate Cox proportional hazard model for primary efficacy outcome. Median age, duration, lesion charactertics, lesion length,critical limb ischemia, history of index-limb revascularization,ischemia stroke, chronic renal failure, pre-procedural medication, post-procedural medication and post-procedural anticoagulant were entered into the multivariate Cox proportional hazard model for secondary efficacy outcome. Diagnosis, lesion length, thrombus debulking, critical limb ischemia,history of index-limb revascularization, hypertension, pre-procedural medication, post-procedural medication, pre-procedural anticoagulant and post-procedural anticoagulant were entered into the multivariate Cox proportional hazard model for principal safety outcome. Median age, CHA_2_DS_2_-VASc score, lesion length, smoking status, ischemia stroke, pre-procedural medication, post-procedural medication, pre-procedural anticoagulant and post-procedural anticoagulant were entered into the multivariate Cox proportional hazard model for secondary safety outcome.

**Table S1. Univariate Cox Regression Analyses of covariates for Primary and Secondary Efficacy Outcomes**

|  | **Primary efficacy outcome**  P Value | **Secondary efficacy outcomes**  P Value | **Major adverse limb events**  P Value | **Death from any cause**  P Value |  |
| --- | --- | --- | --- | --- | --- |
| Median age, years | 0.862 | 0.030 | 0.526 | 0.001 |  |
| Sex | 0.330 | 0.509 | 0.604 | 0.952 |  |
| CHA_2_DS_2_-VASc score | 0.667 | 0.784 | 0.608 | 0.987 |  |
| HAS-BLED score | 0.476 | 0.314 | 0.713 | 0.324 |  |
| Duration | 0.322 | 0.245 | 0.202 | 0.627 |  |
| Diagnosis | 0.251 | 0.843 | 0.079 | 0.142 |  |
| Lesion characteristics | 0.576 | 0.268 | 0.454 | 0.417 |  |
| Lesion length | 0.640 | 0.062 | 0.284 | 0.064 |  |
| Thrombus Debulking | 0.267 | 0.616 | 0.048 | 0.960 |  |
| Critical limb ischemia | 0.095 | 0.033 | 0.210 | 0.106 |  |
| History of index-limb revascularization | 0.311 | 0.102 | 0.656 | 0.162 |  |
| Hypertension | 0.283 | 0.514 | 0.251 | 0.736 |  |
| Diabetes mellitus | 0.722 | 0.699 | 0.574 | 0.178 |  |
| Smoking status | 0.507 | 0.978 | 0.812 | 0.719 |  |
| Coronary artery disease | 0.974 | 0.478 | 0.866 | 0.011 |  |
| Ischemic stroke | 0.696 | 0.279 | 0.690 | 0.058 |  |
| Chronic Renal failure | 0.045 | 0.002 | 0.534 | 0.001 |  |
| Pre-procedural medication | 0.111 | 0.124 | 0.066 | 0.169 |  |
| Post-procedural medication | 0.240 | 0.04 | 0.184 | 0.315 |  |
| Pre-procedural anticoagulant | 0.547 | 0.395 | 0.665 | 0.141 |  |
| Post-procedural anticoagulant | 0.743 | 0.188 | 0.638 | 0.177 |  |

**Table S2. Univariate Cox Regression Analyses of covariates for Safety Outcomes.**

|  | **Principal safety outcome**  P Value | **Secondary safety outcome**  P Value |  |
| --- | --- | --- | --- |
| Median age, years | 0.521 | 0.227 |  |
| Sex | 0.886 | 0.567 |  |
| CHA_2_DS_2_-VASc score | 0.451 | 0.234 |  |
| HAS-BLED score | 0.984 | 0.360 |  |
| Duration | 0.507 | 0.332 |  |
| Diagnosis | 0.782 | 0.906 |  |
| Lesion characteristics | 0.790 | 0.927 |  |
| Lesion length | 0.523 | 0.164 |  |
| Thrombus Debulking | 0.993 | 0.861 |  |
| Critical limb ischemia | 0.892 | 0.728 |  |
| History of index-limb revascularization | 0.420 | 0.976 |  |
| Hypertension | 0.239 | 0.559 |  |
| Diabetes mellitus | 0.438 | 0.675 |  |
| Smoking status | 0.901 | 0.052 |  |
| Coronary artery disease | 0.359 | 0.971 |  |
| Ischemic stroke | 0.717 | 0.136 |  |
| Chronic Renal failure | 0.712 | 0.993 |  |
| Pre-procedural medication | 0.330 | 0.138 |  |
| Post-procedural medication | 0.857 | 0.977 |  |
| Pre-procedural anticoagulant | 0.224 | 0.321 |  |
| Post-procedural anticoagulant | 0.507 | 0.272 |  |
